# A Novel Ophthalmic Solution Containing Glicopro^®^ Complex for the Treatment of Patients with Dry Eye Disease: Results from a Pilot Study

**DOI:** 10.3390/jcm13051447

**Published:** 2024-03-01

**Authors:** Giuseppe Giannaccare, Sabrina Vaccaro, Massimiliano Borselli, Costanza Rossi, Giovanna Carnovale Scalzo, Giovanni Scalia, Lorenzo Di Cesare Mannelli, Carla Ghelardini, Lucrezia Zerillo, Immacolata Polvere, Pasquale Vito, Tiziana Zotti, Romania Stilo, Vincenzo Scorcia

**Affiliations:** 1 Department of Ophthalmology, University Magna Græcia of Catanzaro, 88100 Catanzaro, Italy; sabrina_vaccaro@libero.it (S.V.); mborselli93@gmail.com (M.B.); costanzarossi9@gmail.com (C.R.); giovannacarnovalescalzo@unicz.it (G.C.S.); drscalia.giovanni@gmail.com (G.S.); vscorcia@unicz.it (V.S.); 2 Eye Clinic, Department of Surgical Science, University of Cagliari, 09124 Cagliari, Italy; 3 Department of Neuroscience, Psychology, Drug Research and Child Health-NEUROFARBA-Pharmacology and Toxicology Section, University of Florence, 50139 Florence, Italy; lorenzo.mannelli@unifi.it (L.D.C.M.); carla.ghelardini@unifi.it (C.G.); 4 Department of Science and Technologies, University of Sannio, 82100 Benevento, Italy; lzerillo@unisannio.it (L.Z.); immapolvere88@gmail.com (I.P.); vito@unisannio.it (P.V.); tzotti@unisannio.it (T.Z.); romstilo@unisannio.it (R.S.); 5 Genus Biotech Srls, University of Sannio, 82100 Benevento, Italy

**Keywords:** dry eye disease, ocular surface, tear substitute, proenkephalin, GlicoPro^®^, ocular discomfort

## Abstract

(1) **Background**: Dry eye disease (DED) is a multifactorial ocular surface disease characterized by an imbalance in ocular surface homeostasis, and tear substitutes constitute the first line of treatment. The present study aimed to evaluate the changes in the signs and symptoms of patients with DED treated with a novel tear substitute containing the GlicoPro^®^ complex. (2) **Methods**: Patients with DED not successfully responding to other tear substitutes were enrolled and treated with a novel ophthalmic solution (two drops four times daily). Patients were examined before starting the study treatment (T0) and after 30 (T1) and 60 (T2) days of treatment by means of Keratograph for the evaluation of the following: (i) tear meniscus height (TMH); (ii) noninvasive Keratograph break-up time (NIKBUT); (iii) bulbar redness; and (iv) infrared meibography. The SANDE questionnaire was administered to assess ocular discomfort symptoms. Analysis of the tear content of proenkephalin and Met/Leu-enkephalin was also performed. (3) **Results**: At T2, a significant improvement in NIKBUT first, average, and class, TMH, and SANDE score was found. The tear content of proenkephalins was significantly higher at T1, whereas processed active Met/Leu-enkephalins increased at both T1 and T2. (4) **Conclusions**: Our novel tear substitute based on GlicoPro^®^ resulted in a significant improvement in ocular discomfort symptoms, tear volume, and stability in the patients treated. The increase in active peptides processed in tears may represent the pathophysiological substrate underlying this finding.

## 1. Introduction

Dry eye disease (DED) is a complex multifactorial ocular surface disease characterized by an imbalance in ocular surface homeostasis and characterized by ocular symptoms [1]. As highlighted in the definition of the International Dry Eye Workshop (DEWS II), inflammation, ocular surface damage, and neurosensory abnormalities play an important etiologic role [2]. Risk factors for diseases, specifically in DED, can be categorized into several groups based on their nature and impact on risk. Clinical risk factors include anterior and posterior blepharitis and the use of topical and systemic medications. Environmental factors involve adverse conditions and the prolonged use of video display terminals. Behavioral factors entail the use of contact lenses. Medical factors encompass ocular surgery and autoimmune diseases. Lastly, biological factors include predisposing ocular anatomical abnormalities, female gender, Asian race, and advanced age [3,4]. The two primary categories of DED are evaporative and aqueous-deficient. Evaporative DED is primarily associated with alterations in the meibomian glands, while aqueous-deficient DED is caused by reduced tear production. Evaporative DED is considered more common than the aqueous deficiency form [2]. The eye surface is covered by the tear film, which is composed of three distinct layers: the lipid layer produced by meibomian glands, the aqueous layer produced by lacrimal glands, and the mucin layer produced by goblet cells. The tear film is adequately distributed over the ocular surface during blinking [5]. Evaporation of the aqueous component of the tear film and the resulting instability lead to a condition of tear hyperosmolarity, which is one of the central mechanisms of DED. Hyperosmolarity is capable of triggering the activation of the inflammatory cascade that leads to cellular damage, particularly through the loss of conjunctival cells that produce mucin, which further exacerbates tear film instability and creates a vicious cycle [6,7,8]. It is well established that inflammation and immune dysregulation are key etiological factors in the pathogenesis of DED. Monocytes, macrophages, and neutrophils directly regulate proinflammatory stimuli, increasing levels of Tumor Necrosis Factor-alpha (TNF-α), Interleukin-1 (IL-1), Interleukin-6 (IL-6), Interleukin-8 (IL-8), and other cytokines in conditions of corneal desiccation and ocular surface alterations in the contest of DED [9]. Moreover, in the conjunctiva and corneal epithelium in DED patients, other inflammatory markers such as human leukocyte antigen (HLA)-DR [7] and chemokine (C-C motif) ligand 2 (CCL2) [10] have been found. Lam et al. [11] showed that in patients with DED, IL-6, IL-8, and TNF-α were significantly increased compared with the controls. In particular, IL-6 and irritation symptom severity were significantly correlated, suggesting that IL-6 may be the result of neuropathic eye pain [8]. Patient-reported symptoms include burning, foreign body sensation, and ocular discomfort, which include dryness, irritation, grinding, scratching, sanding sensation, soreness, stinging, burning, itching, and eye fatigue up to ocular pain, compromising quality of life and work productivity, and thus posing a serious public health problem [12,13,14,15]. 

Another issue exacerbating eye pain depends on the physiological levels of enkephalins, natural analgesic peptides playing a pivotal role in pain modulation [16]. In the Central Nervous System (CNS), they interact with opioid receptors, thereby attenuating pain transmission along nerve pathways. However, the analgesic effects of enkephalins are often short-lived and localized due to their rapid degradation by neprilysin neutral endopeptidase (NEP) and aminopeptidase N (APN) [17]. Recently, a novel therapeutic approach has been proposed for the control of discomfort/pain symptoms focused on opiorphin, an endogenous peptide with potent analgesic properties thanks to its ability to protect enkephalins from degradation [18]. Opiorphin is one of the major endogenous metabolites and is secreted in tears, where its concentration increases in response to pain [19]. A new ophthalmic solution (Lacricomplex^®^, FB Vision, Ascoli Piceno, Italy) containing GlicoPro^®^ (FB Vision, Ascoli Piceno, Italy), a multimolecular complex based on proteins, sulfured and unsulfured glycosaminoglycans (GAGs)—useful for lubricating, stabilizing tear film, and prolonging pre-corneal persistence—and opiorphin, which assists the physiological pain-relieving mechanism by enhancing Met/Leu-enkephalin concentrations, has recently become commercially available [20,21,22,23]. In DED corneal tissues treated with GlicoPro^®^, histo-morphologic analysis demonstrated restoration of the corneal epithelium, microvilli, and mucin network [20].

The purpose of this study was to evaluate the changes in subjective symptoms and objective signs occurring in patients suffering from DED, not responding to other tear substitutes, treated with Lacricomplex^®^. In addition, the tear concentration of enkephalins and their precursor protein, proenkephalin, has also been analyzed.

## 2. Materials and Methods

### 2.1. Study and Patients

In this prospective pilot study, patients with DED already diagnosed according to the TFOS DEWS II criteria [24] were examined and screened for enrolment at a tertiary referral center (Department of Ophthalmology, University of Magna Grecia, Catanzaro, Italy) between March 2022 and December 2022. The study was approved by the local ethics committee (Comitato Etico Regione Calabria Sezione Area Centro—N.167, 22 April 2021). Detailed informed consent for participation in the study was signed by all patients, in accordance with the 1964 Declaration of Helsinki. The inclusion criterion for patient enrollment was a diagnosis of DED, with noninvasive breakup time (NIBUT) less than 10 s and a pathological value of the Symptom Assessment in Dry Eye (SANDE) questionnaire, not successfully controlled with other tear substitutes. Patients were excluded if one or more of the following conditions were present: recent (<3 months) ocular surgery, systemic disease or therapies affecting tear secretion, concomitant ocular diseases, or concomitant use of other topical medications (e.g., corticosteroids, nonsteroidal anti-inflammatory drugs). Patients who satisfied the study criteria were enrolled and treated in both eyes with Lacricomplex^®^ according to the following therapeutic regimen: 2 drops 4 times daily for 60 days.

### 2.2. Ocular Surface Workup

All patients underwent noninvasive examination of the ocular surface using Keratograph 5M (Oculus, Wetzlar, Germany) before starting treatment (T0) and 30 ± 2 days (T1) and 60 ± 4 days (T2) after treatment for the evaluation of the following: (i) tear meniscus height (TMH); (ii) noninvasive Keratograph break-up time (NIKBUT) (a) first, (b) average, and (c) class (0:>10 s [s]; I: 6–10 s; II 3–6 s; III< 3 s); (iii) bulbar redness; and (iv) infrared meibography for evaluating meibomian glands loss (MGL). Measurements were taken in a dimly lit room during a single visit. Temperature was maintained between 21 and 24 °C, and humidity was controlled within the range of 30–60%. The TMH, NIKBUT first, NIKBUT average, and ocular redness were automatically measured using Oculus Keratograph 5M software (v. 2.15r2), following the instructions provided by the supplier of the equipment. For each participant, lower TMH pictures were captured and measured perpendicular to the lid margin at the central position relative to the pupil center using an included ruler [25]. The NIKBUT was quantified as the duration in seconds between the final full blink to the initial disruption of placid rings projected onto the cornea’s surface, which was automatically detected by the device. The instrument produced two metrics for NIKBUT: the amount of time until the initial disruption of the tear film (NIKBUT-first) and the mean duration of all rupture occurrences (NIKBUT-average) [26]. To evaluate the level of gland deficiency, infrared meibography was conducted on both the upper and lower eyelids. This involved using a grading system called meiboscore, which categorizes deficiency on a scale of 0 to 3. More precisely, grade 0 signifies the complete absence of gland loss, grade 1 indicates gland loss affecting up to 33% of the total gland area, grade 2 represents gland loss ranging from 33% to 66%, and grade 3 denotes gland loss of 67% or more [27,28,29]. Bulbar redness measurements were automatically obtained using the Oculus Keratograph 5M software [27,30]. Symptoms of ocular discomfort were assessed using the SANDE questionnaire. The SANDE questionnaire consists of two questions assessing the frequency and severity of dry eye syndrome. This survey employs a 100 mm horizontal line for each question to evaluate the level of ocular discomfort and/or dryness reported by the patients. The questionnaire assesses the frequency of symptoms on a scale that runs from “rarely” to “all of the time”, and the intensity of symptoms runs on a scale that ranges from “very mild” to “very severe”. The positions of the patients’ marks on the 100 mm horizontal lines were measured in millimeters from left to right and documented. The data acquired from the SANDE questionnaire were utilized for determining the result by multiplying the frequency score with the severity score and, after that, obtaining the square root [31,32].

### 2.3. Tear Analysis

In a subgroup of patients with an adequate amount of tears for the collection (TMH value ≥ 0.25 mm [mm] at T0), a sample of 10 μL of tears was obtained to evaluate the con-centration of proenkephalin and Met/Leu-enkephalin. The method that was utilized to choose this particular subgroup in the study was based on voluntary participation. Briefly, tears collected by capillary tube were blown into microcentrifuge tubes, stored at −80 °C, and used within 2 months. The total protein amount in the tears was determined by performing a Bradford Assay according to the manufacturer’s recommendations (Protein assay Dye reagent concentrate, Bio-Rad, Hercules, CA, USA). The absorbance was measured at 595 nm with a spectrophotometer (NanoDrop One, Thermo Scientific™, Waltham, MA, USA), and the protein concentrations were derived using a bovine serum albumin (BSA) calibration curve [33]. Thirty-five micrograms of tear proteins was used for Western blot analyses, and ten micrograms was used for Coomassie blue staining. Separation by SDS-PAGE, blotting, and incubation with primary and secondary antibodies were performed as described elsewhere [34]. A chemiluminescence reaction was carried out with Clarity Western ECL Substrate (Bio-Rad Laboratories, Inc., Hercules, CA, USA), and signal acquisition was performed through the ChemiDoc Xrs+ by Image Lab software (v 1.4.3.67) (Bio-Rad Laboratories, Inc., Hercules, CA, USA). The antisera and monoclonal antibodies used in the present work are as follows: HRP-conjugated anti-mouse (Sigma Aldrich, Saint Louis, MO, USA) and anti-human Met/Leu-enkephalin (cat. No. sc-47705; Santa Cruz Biotechnology, Santa Cruz, CA, USA). In the Coomassie blue staining following SDS-PAGE, the polyacrylamide gel was stained with a staining solution (0.4% Coomassie blue, 50% methanol, 10% acetic acid) for 30 min at room temperature. The gel was sequentially soaked into a destaining solution-I (50% methanol, 10% acetic acid) for 30–60 min until the protein bands were discretely observable [35]. Signals were analyzed by ImageJ software (v 1.4.3.67).

### 2.4. Outcomes

The primary outcome was the changes in objective signs and subjective symptoms occurring after the study treatment. The secondary outcome was the change in tears content (proenkephalin and Met/Leu-enkephalin) registered after treatment in a subgroup of patients with an adequate amount of tears for collection.

### 2.5. Statistical Analysis

Statistical analysis was performed using Prism version 9.4.0 (GraphPad Software Inc., San Diego, CA, USA). Normally distributed data were expressed as mean ± standard deviation (SD); otherwise, they were expressed as median values with interquartile range (IQR). Parametric and nonparametric tests were chosen on the basis of data normality. The D’Agostino and Pearson test and the Shapiro–Wilk test were applied to assess if data were normally distributed. TMH (*p* < 0.001; *p* < 0.001), NIKBUT first (*p* < 0.001; *p* < 0.001), NIKBUT class (*p* < 0.001; *p* < 0.001), bulbar redness (*p* < 0.001; *p* < 0.001), MGL (*p* = 0.038; *p* < 0.001), and SANDE (*p* = 0.106; *p* < 0.001) did not pass the D’Agostino and Pearson test and the Shapiro–Wilk test. However, the NIKBUT average (*p* = 0.196; *p* < 0.065) passed the D’Agostino and Pearson test and the Shapiro–Wilk test. SANDE (*p* = 0.106) passed the D’Agostino and Pearson test.

Student’s *t*-test, the Mann–Whitney U test, Dunnett’s multiple comparison test, and the Friedman test were used to compare variables when appropriate. A *p*-value <0.05 was considered statistically significant. To determine the sample size of the study, a priori power analysis was performed based on the data of the study of Lambiase and collaborators [36]. In total, 19 patients were required to detect a mean change in SANDE from the baseline of 16.1 points, with a power of 0.95 and a *p* value of 0.05.

## 3. Results

A total of 60 patients (23 males, 37 females; mean age 67.00 ± 8.00 years) with DED were included in the study. Baseline demographic and clinical characteristics of the enrolled patients are summarized in Table 1.

### 3.1. Ocular Parameters

**Figure 1:**
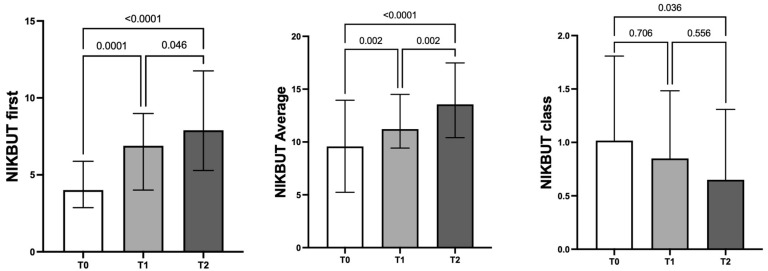
Effect of Lacricomplex^®^ on NIKBUT first, average, and NIBUT class. Both NIKBUT first and average significantly increased at T1 and T2 versus T0. With respect to baseline, NIKBUT class significantly improved 2 months after the baseline (T0).

At both T1 and T2, a significant improvement in NIKBUT first (from 4.01 [2.87–5.88] s to 6.89 [4.01–8.98] s [Friedman test; *p* = 0.0001] and 7.90 [5.28–11.76] s [Friedman test; *p* < 0.0001], respectively) and NIKBUT average (from 9.63 ± 5.03 s to 11.72 ± 3.84 s [one-way ANOVA; *p* = 0.002] and 13.85 ± 4.88 s [one-way ANOVA; *p* < 0.0001], respectively) was found; NIKBUT class showed a significant improvement at T2 (from 1.00 [0.00–2.00] to 1.00 [0.00–1.00] [Friedman test; *p* = 0.036]) (Figure 1). 

**Figure 2:**
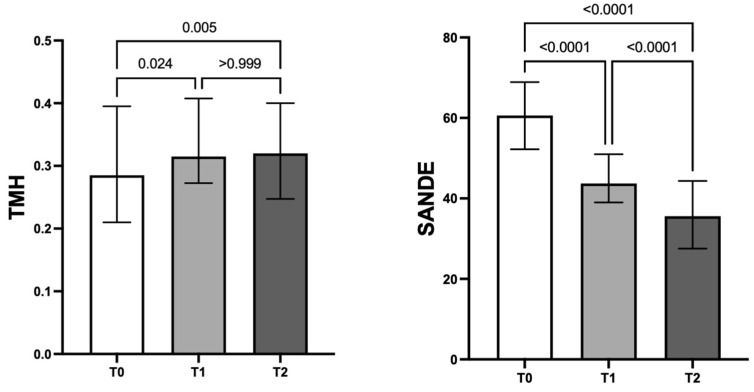
Effect of a new tear substitute based on GlicoPro^®^ on TMH and SANDE score. With respect to baseline, TMH value significantly increased 1 and 2 months after the baseline (T0) (*p* = 0.024; *p* = 0.005); conversely, there were no statistically significant differences from T1 to T2 (*p* > 0.999). With respect to baseline, SANDE score significantly decreased at both study time points, namely 1 and 2 months after the baseline (T0) (*p* < 0.0001).

The mean value of the TMH increased significantly from T0 to both T1 and T2 (from 0.28 [0.21–0.39] mm at T0 to 0.31 [0.27–0.40] [Friedman test; *p* = 0.024] and 0.32 [0.24–0.40] [*p* = 0.005], respectively). SANDE score significantly decreased from a baseline value of 60.60 (52.21–68.90) to 43.72 (39.00–50.98) (one-way ANOVA; *p* < 0.0001) at T1 and 35.60 (27.53–44.33) (one-way ANOVA; *p* < 0.0001) at T2 (Figure 2).

Conversely, no statistically significant reduction was detected at each time point for bulbar redness (from 1.35 [1.02–1.60] at T0 to 1.30 [1.00–1.77] and 1.20 [1.00–1.60] [Friedman test; *p* > 0.999], respectively) and MGL (from 1.50 [1.00–2.00] at T0 to 2.00 [1.00–2.00] and 2.00 [1.00–2.00] [Friedman test; *p* > 0.999], respectively). No adverse events related to the use of the study treatment were reported during the entire period.

### 3.2. Tear Analysis

**Figure 3:**
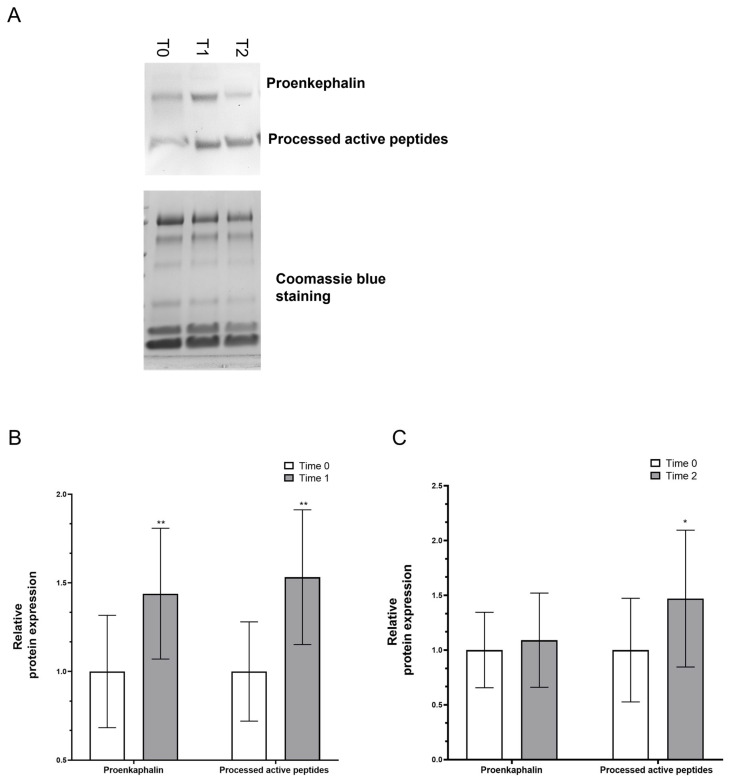
(**A**) Representative immunoblotting of proenkephalins and processed active peptides in tear samples. Coomassie blue staining was used as the loading control (* *p* < 0.05; ** *p* < 0.01). (**B**) Proenkephalin and processed active peptides levels significantly increased at T1 with respect to T0 (*p* = 0.005 and *p* < 0.001, respectively). (**C**) After 60 days (T2), only processed active peptide level remained higher compared to T0 (*p* = 0.048).

In nine patients, the levels of lacrimal proenkephalins and processed active peptides, namely Met/Leu-enkephalin, were evaluated using an immunoblot assay (Figure 3A). Notably, the densitometric analysis of the Western blots indicated that at T1, both proenkephalins and processed active peptides significantly increased with respect to those measured at T0 from 1 ± 0.63 to 1.43 ± 0.73 (*t*-test *p* = 0.005) and from 1 ± 0.56 to 1.47 ± 1.25 (*t*-test *p* < 0.001), respectively (Figure 3B). Differently, at T2, that is after 60 days of daily treatment, only the processed active peptide level remained higher compared to T0 (1.53 ± 0.76 respect to 1 ± 0.95; *t*-test *p* = 0.048), whereas proenkephalins were restored to their initial conditions (1.09 ± 0.86 respect to 1 ± 0.69; *t*-test *p* = 0.277) (Figure 3C). 

## 4. Discussion

The present study reports the preliminary results of the first pilot study investigating the effects of a novel ophthalmic preparation based on GlicoPro^®^, a multimolecular complex with lubricating, moisturizing, antioxidant, and protective effects. After 2 months of treatment, the NIKBUT (both first and average) and TMH improved significantly at each time point; in parallel, the ocular discomfort symptoms evaluated by SANDE score reduced significantly. Clinical outcomes were further supported by the molecular changes detected in the tear fluid of patients who received the treatment. Consistently, an increase in the ratio between processed active enkephalins and their precursor proenkephalins was observed at T2 with respect to T1, suggesting that the bioavailability of opioid receptor ligands persists in the tears of treated eyes.

A previous in vitro study showed that this product contributes to the physiological repair processes at the corneal level, supporting the restoration of the functionality of the corneal nerve terminations and the process of corneal healing with a significant inhibitory effect on enkephalinase enzymes [10]. Therefore, the increase in tear active proenkephalins observed in the treated eyes could be mainly ascribed to opiorphin, a natural peptide contained in the protein fraction of GlicoPro^®^, which is known to assist the physiological pain-relieving mechanism of the eye. Opiorphin is endogenously present in body fluids, primarily in tears, whose level is enhanced in pathological and painful conditions. Salivary opiorphin is increased in patients with burning mouth syndrome [37], as well as in patients with dental pain caused by pulp inflammation [38]. In tears, opiorphin levels increase in the presence of ocular pain caused by a corneal foreign body [19]. The role of opiorphin in pain modulation has been extensively described [19,39,40,41]. This pentapeptide enhances endogenous opioid signaling by protecting enkephalins metabolism. Opiorphin is an inhibitor of the enzymes neprilysin (neutral endopeptidase) and aminopeptidase N; consequently, the Met/Leu-enkephalin concentration increases [39,42,43]. The pharmacodynamic peculiarity makes opiorphin a physiological pain modulator able to counteract nociceptive and neuropathic pain-reducing hypersensitivity with an opioid receptor-dependent mechanism [44,45]. The safety profile of opiorphin is significantly better than direct opioid agonists since its effects depend on the concentration of enkephalins released in response to a painful stimulus rather than direct receptor action [16].

The observed enhanced concentration of Met/Leu-enkephalin by about 50% suggests the relevance of opioid signaling in the relief of ocular discomfort. It should also be noted that repeated treatment with GlicoPro^®^ increased the enkephalin precursor proenkephalin in tears only at T1, suggesting that the opiorphin-induced modulation of opioid signaling could be mainly due to its catabolism reduction activity rather than the anabolism enhancement of enkephalins. However, we decided to use noninvasive diagnostic workup, while the evaluation of invasive tear break-up time (TBUT) involves the application of fluorescein to the eye. At the same time, we decided to collect tear samples to study pathophysiological pathways in particular to measure the concentration of proenkephalins and Met/Leu-enkephalin.

The present pilot study suffers from some limitations that deserve mentioning. First, the study design lacks a control group of patients treated with a placebo or vehicle. Due to the absence of a control group, we considered utilizing patients who did not respond to other tear substitutes. However, it is important to note that all types of dry eye could benefit from this treatment. In this study, thanks to the switch to a new formulation, patients were able to better manage ocular discomfort symptoms, obtaining a concomitant improvement in their tear film stability and volume. Second, the study was not powered to detect differences in tear content; thus, the lack of significancy of the proenkephalin variation at T2 could be related to the small sample size of the subgroup of patients with an adequate tear volume required for collection, avoiding reflex tearing.

## 5. Conclusions

In conclusion, topical treatment with a novel GlicoPro^®^-based tear substitute significantly improved ocular discomfort symptoms and objective signs in patients with DED who did not respond to other tear substitutes. These findings suggest a special ability of the new product thanks to the multi-target approach allowed due to its complex composition.

## Figures and Tables

**Table 1:** Baseline patient characteristics.

Age, mean value (SD), years	67.00 (8.00)
Sex (M/F)	23/37
Caucasian race, *n* (%)	60 (100)
TMH, median value (IQR), mm	0.28 (0.21–0.39)
NIKBUT first, median value (IQR), s	4.01 (2.87–5.88)
NIKBUT average, mean value (SD), s	9.63 (5.03)
SANDE score, median value (IQR)	60.60 (52.21–68.90)
Bulbar redness score, median value (IQR)	1.35 (1.02–1.60)
MGL scale, median value (IQR)	1.50 (1.00–2.00)

Abbreviations: SD, standard deviation; M/F, male/female; IQR, interquartile range; TMH, tear meniscus height; NIKBUT, noninvasive Keratograph break-up time; SANDE, Symptom Assessment in Dry Eye; MGL, meibomian glands loss; mm, millimeters; s, seconds.

## Data Availability

The raw data supporting the conclusions of this article will be made available by the authors without undue reservation.

## References

[1] Rouen P.A., White M.L. (2018). Dry Eye Disease: Prevalence, Assessment, and Management. Home Healthc. Now.

[2] Craig J.P., Nichols K.K., Akpek E.K., Caffery B., Dua H.S., Joo C.K., Liu Z., Nelson J.D., Nichols J.J., Tsubota K. (2017). TFOS DEWS II Definition and Classification Report. Ocul. Surf..

[3] Bron A.J., de Paiva C.S., Chauhan S.K., Bonini S., Gabison E.E., Jain S., Knop E., Markoulli M., Ogawa Y., Perez V. (2017). TFOS DEWS II pathophysiology report. Ocul. Surf..

[4] Stapleton F., Alves M., Bunya V.Y., Jalbert I., Lekhanont K., Malet F., Na K.S., Schaumberg D., Uchino M., Vehof J. (2017). TFOS DEWS II Epidemiology Report. Ocul. Surf..

[5] Sridhar M.S. (2018). Anatomy of cornea and ocular surface. Indian J. Ophthalmol..

[6] Luo L., Li D.Q., Corrales R.M., Pflugfelder S.C. (2005). Hyperosmolar saline is a proinflammatory stress on the mouse ocular surface. Eye Contact Lens..

[7] Li D.Q., Chen Z., Song X.J., Luo L., Pflugfelder S.C. (2004). Stimulation of matrix metalloproteinases by hyper-osmolarity via a JNK pathway in human corneal epithelial cells. Investig. Ophthalmol. Vis. Sci..

[8] Rhee M.K., Mah F.S. (2017). Inflammation in Dry Eye Disease: How Do We Break the Cycle?. Ophthalmology.

[9] Nair A.P., D’Souza S., Shetty R., Ahuja P., Kundu G., Khamar P., Dadachanji Z., Paritekar P., Patel P., Dickman M.M. (2021). Altered ocular surface immune cell profile in patients with dry eye disease. Ocul. Surf..

[10] Na K.S., Mok J.W., Kim J.Y., Rho C.R., Joo C.K. (2012). Correlations between tear cytokines, chemokines, and soluble receptors and clinical severity of dry eye disease. Investig. Ophthalmol. Vis. Sci..

[11] Lam H., Bleiden L., de Paiva C.S., Farley W., Stern M.E., Pflugfelder S.C. (2009). Tear cytokine profiles in dysfunctional tear syndrome. Am. J. Ophthalmol..

[12] Johnson M.E. (2009). The association between symptoms of discomfort and signs in dry eye. Ocul. Surf..

[13] Friedman N.J. (2010). Impact of dry eye disease and treatment on quality of life. Curr. Opin. Ophthalmol..

[14] Aragona P., Giannaccare G., Mencucci R., Rubino P., Cantera E., Rolando M. (2021). Modern approach to the treatment of dry eye, a complex multifactorial disease: A P.I.C.A.S.S.O. board review. Br. J. Ophthalmol..

[15] Barabino S., Labetoulle M., Rolando M., Messmer E.M. (2016). Understanding Symptoms and Quality of Life in Patients with Dry Eye Syndrome. Ocul. Surf..

[16] Reaux-Le Goazigo A., Poras H., Ben-Dhaou C., Ouimet T., Baudouin C., Wurm M., Parsadaniantz S.M. (2019). Dual enkephalinase inhibitor PL265: A novel topical treatment to alleviate corneal pain and inflammation. Pain.

[17] Kanjhan R. (1995). Opioids and pain. Clin. Exp. Pharmacol. Physiol..

[18] Giannaccare G., Ghelardini C., Mancini A., Scorcia V., Di Cesare Mannelli L. (2021). New Perspectives in the Pathophysiology and Treatment of Pain in Patients with Dry Eye Disease. J. Clin. Med..

[19] Ozdogan S., Sonmez C., Yolcu D., Gungormus M. (2020). Tear Opiorphin Levels in Ocular Pain Caused by Corneal Foreign Body. Cornea.

[20] Mencucci R., Strazzabosco G., Cristofori V., Alogna A., Bortolotti D., Gafà R., Cennamo M., Favuzza E., Trapella C., Gentili V. (2021). GlicoPro, Novel Standardized and Sterile Snail Mucus Extract for Multi-Modulative Ocular Formulations: New Per-spective in Dry Eye Disease Management. Pharmaceutics.

[21] Trapella C., Rizzo R., Gallo S., Alogna A., Bortolotti D., Casciano F., Zauli G., Secchiero P., Voltan R. (2018). HelixComplex snail mucus exhibits pro-survival, proliferative and pro-migration effects on mammalian fibroblasts. Sci. Rep..

[22] Tsoutsos D., Kakagia D., Tamparopoulos K. (2009). The efficacy of Helix aspersa Müller extract in the healing of partial thickness burns: A novel treatment for open burn management protocols. J. Dermatolog. Treat..

[23] Gentili V., Bortolotti D., Benedusi M., Alogna A., Fantinati A., Guiotto A., Turrin G., Cervellati C., Trapella C., Rizzo R. (2020). HelixComplex snail mucus as a potential technology against O3 induced skin damage. PLoS ONE.

[24] Wolffsohn J.S., Arita R., Chalmers R., Djalilian A., Dogru M., Dumbleton K., Gupta P.K., Karpecki P., Lazreg S., Pult H. (2017). TFOS DEWS II diagnostic methodology report. Ocul. Surf..

[25] García-Montero M., Rico-Del-Viejo L., Lorente-Velázquez A., Martínez-Alberquilla I., Hernández-Verdejo J.L., Madrid-Costa D. (2019). Repeatability of Noninvasive Keratograph 5M Measurements Associated with Contact Lens Wear. Eye Contact Lens..

[26] Tian L., Qu J.H., Zhang X.Y., Sun X.G. (2016). Repeatability and Reproducibility of Noninvasive Keratograph 5M Measurements in Patients with Dry Eye Disease. J. Ophthalmol..

[27] Rico-Del-Viejo L., Benítez-Del-Castillo J.M., Gómez-Sanz F.J., García-Montero M., Llorens-Quintana C., Madrid-Costa D. (2019). The influence of meibomian gland loss on ocular surface clinical parameters. Cont. Lens Anterior Eye.

[28] Garcia-Queiruga J., Pena-Verdeal H., Sabucedo-Villamarin B., Garcia-Resua C., Giraldez M.J., Yebra-Pimentel E. (2023). Analysis of the Differences in Ocular Surface Damage and Inflammatory Signs between Healthy and Evaporative Dry Eye Participants. Ocul. Immunol. Inflamm..

[29] Pult H., Riede-Pult B.H. (2012). Non-contact meibography: Keep it simple but effective. Cont. Lens Anterior Eye.

[30] Schulze M.M., Ng A., Yang M., Panjwani F., Srinivasan S., Jones L.W., Senchyna M. (2021). Bulbar Redness and Dry Eye Disease: Comparison of a Validated Subjective Grading Scale and an Objective Automated Method. Optom. Vis. Sci..

[31] Gulati A., Sullivan R., Buring J.E., Sullivan D.A., Dana R., Schaumberg D.A. (2006). Validation and repeatability of a short questionnaire for dry eye syndrome. Am. J. Ophthalmol..

[32] Schaumberg D.A., Gulati A., Mathers W.D., Clinch T., Lemp M.A., Nelson J.D., Foulks G.N., Dana R. (2007). Development and validation of a short global dry eye symptom index. Ocul. Surf..

[33] Kielkopf C.L., Bauer W., Urbatsch I.L. (2020). Bradford Assay for Determining Protein Concentration. Cold Spring Harb. Protoc..

[34] Mazzone P., Congestrì M., Scudiero I., Polvere I., Voccola S., Zerillo L., Telesio G., Vito P., Stilo R., Zotti T. (2020). UBAC1/KPC2 regulates TLR3 signaling in human keratinocytes through functional interaction with the CARD14/CARMA2sh-TANK complex. Int. J. Mol. Sci..

[35] Brunelle J.L., Green R. (2014). Coomassie blue staining. Methods Enzymol..

[36] Lambiase A., Sullivan B.D., Schmidt T.A., Sullivan D.A., Jay G.D., Truitt E.R., Bruscolini A., Sacchetti M., Mantelli F. (2017). A Two-Week, Randomized, Double-masked Study to Evaluate Safety and Efficacy of Lubricin (150 μg/mL) Eye Drops Versus Sodium Hyaluronate (HA) 0.18% Eye Drops (Vismed®) in Patients with Moderate Dry Eye Disease. Ocul. Surf..

[37] Salarić I., Sabalić M., Alajbeg I. (2017). Opiorphin in burning mouth syndrome patients: A case-control study. Clin. Oral Investig..

[38] Ozdogan M.S., Gungormus M., Ince Yusufoglu S., Ertem S.Y., Sonmez C., Orhan M. (2019). Salivary opiorphin in dental pain: A potential biomarker for dental disease. Arch. Oral Biol..

[39] Dufour E., Villard-Saussine S., Mellon V., Leandri R., Jouannet P., Ungeheuer M.N., Rougeot C. (2013). Opiorphin se-cretion pattern in healthy volunteers: Gender difference and organ specificity. Biochem. Anal. Biochem..

[40] Wisner A., Dufour E., Messaoudi M., Nejdi A., Marcel A., Ungeheuer M.N., Rougeot C. (2006). Human Opiorphin, a natural antinociceptive modulator of opioid-dependent pathways. Proc. Natl. Acad. Sci. USA.

[41] Mennini N., Mura P., Nativi C., Richichi B., Di Cesare Mannelli L., Ghelardini C. (2015). Injectable liposomal formulations of opiorphin as a new therapeutic strategy in pain management. Future Sci. OA.

[42] Rougeot C., Messaoudi M. (2007). Identification of human opiorphin, a natural antinociceptive modulator of opioid dependent pathways. Med. Sci..

[43] Power I. (2011). An update on analgesics. Br. J. Anaesth..

[44] Popik P., Kamysz E., Kreczko J., Wrobel M. (2010). Human opiorphin: The lack of physiological dependence, tolerance to antinociceptive effects and abuse liability in laboratory mice. Behavioural Brain Res..

[45] Rougeot C., Robert F., Menz L., Bisson J.F., Messaoudi M. (2010). Systemically active human opiorphin is a potent yet non-addictive analgesic without drug tolerance effects. J. Physiol. Pharmacol..

